# PAD4 Inhibitor‐Functionalized Layered Double Hydroxide Nanosheets for Synergistic Sonodynamic Therapy/Immunotherapy Of Tumor Metastasis

**DOI:** 10.1002/advs.202401064

**Published:** 2024-05-06

**Authors:** Di Zhu, Yu Lu, Shuqing Yang, Tingting Hu, Chaoliang Tan, Ruizheng Liang, Yuji Wang

**Affiliations:** ^1^ Department of Medicinal Chemistry College of Pharmaceutical Sciences of Capital Medical University Beijing 100069 P. R. China; ^2^ State Key Laboratory of Chemical Resource Engineering Beijing Advanced Innovation Center for Soft Matter Science and Engineering Beijing University of Chemical Technology Beijing 100029 P. R. China; ^3^ Department Electrical and Electronic Engineering The University of Hong Kong Pokfulam Road Hong Kong SAR 999077 P. R. China; ^4^ Quzhou Institute for Innovation in Resource Chemical Engineering Quzhou 324000 P. R. China; ^5^ Beijing Area Major Laboratory of Peptide and Small Molecular Drugs Engineering Research Center of Endogenous Prophylactic of Ministry of Education of China Beijing Laboratory of Biomedical Materials, Laboratory for Clinical Medicine, Capital Medical University Beijing Laboratory of Oral Health Beijing 100069 P. R. China

**Keywords:** immunogenic cell death, immunotherapy, layered double hydroxides, sonodynamic therapy, tumor metastasis

## Abstract

Sonodynamic therapy (SDT) is demonstrated to trigger the systemic immune response of the organism and facilitate the treatment of metastatic tumors. However, SDT‐mediated neutrophil extracellular traps (NETs) formation can promote tumor cell spread, thus weakening the therapeutic effectiveness of metastatic tumors. Herein, the amorphous CoW‐layered double hydroxide (a‐CoW‐LDH) nanosheets are functionalized with a peptidyl arginine deiminase 4 (PAD4) inhibitor, i.e., YW3‐56, to construct a multifunctional nanoagent (a‐LDH@356) for synergistic SDT/immunotherapy. Specifically, a‐CoW‐LDH nanosheets can act as a sonosensitizer to generate abundant reactive oxygen species (ROS) under US irradiation. After loading with YW3‐56, a‐LDH@356 plus US irradiation not only effectively induces ROS generation and immunogenic cell death, but also inhibits the elevation of citrullinated histone H3 (H3cit) and the release of NETs, enabling a synergistic enhancement of anti‐tumor metastasis effect. Using 4T1 tumor model, it is demonstrated that combining a‐CoW‐LDH with YW3‐56 stimulates an anti‐tumor response by upregulating the proportion of immune‐activated cells and inducing polarization of M1 macrophages, and inhibits immune escape by downregulating the expression of PD‐1 on immune cells under US irradiation, which not only arrests primary tumor progression with a tumor inhibition rate of 69.5% but also prevents tumor metastasis with the least number of lung metastatic nodules.

## Graphical abstract

The a‐CoW‐LDH nanosheets are employed to load YW3‐56 to construct a multifunctional nanoagent (a‐LDH@356) for synergistic SDT/immunotherapy, which effectively induces ROS generation and ICD and inhibits the elevation of H3cit and the release of NETs, synergistically suppressing tumor growth with a tumor inhibition rate of 69.5% and inhibiting tumor metastasis with the least number of lung metastatic nodules.

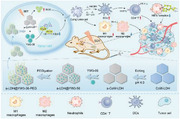

## 1 Introduction

Tumor metastasis accounts for ≈90% of all cancer‐associated deaths.^[^ ^1^ ^−^ ^4^ ^]^ The migration and invasion of tumor cells are important prognostic factors that affect the effectiveness of tumor treatment and the long‐term survival of patients. Compared with primary tumors, tumor metastasis makes it hard to undergo clinical surgical resection or drug treatment because of their high heterogeneity, small size, and wide distribution.^[^ ^5^, ^6^ ^]^ Over the past few decades, various cancer treatment modalities (e.g., chemotherapy, radiotherapy, photothermal therapy, chemodynamic therapy, gas therapy, etc.) have been optimized to eradicate primary tumors but are not effective in treating tumor metastasis. Therefore, it is extremely urgent to develop new strategies for preventing or treating tumor metastasis. Harnessing the immune system to kill cancer cells in blood circulation or metastatic sites has been proven to be a powerful strategy for anti‐tumor metastasis. Several cancer treatment modalities have shown the ability to induce immune response, such as sonodynamic therapy (SDT), photodynamic therapy, and immunotherapy.^[^ ^7^ ^−^ ^11^ ^]^ Among them, SDT has generated a groundswell of research owing to its high controllability, deep tissue penetration depth, tumor‐specific, and noninvasiveness, which employs ultrasound (US) to activate sonosensitizers to trigger the generation of reactive oxygen species (ROS) for inducing excessive oxidative stress and promoting cancer cell death via pathways of apoptosis and/or necrosis.^[^ ^12^ ^−^ ^15^ ^]^ Moreover, sonosensitizers are capable of triggering the systemic immune response of the organism through immunogenic cell death (ICD), facilitating the treatment of metastatic tumors.^[^ ^16^, ^17^ ^]^ However, the SDT‐mediated immune response leads to the formation of neutrophil extracellular traps (NETs), which can promote tumor cell spread and disease progression by promoting tumor cell metabolism, thus weakening the therapeutic effectiveness of metastatic tumors.^[^ ^10^, ^18^ ^]^ Additionally, immunosuppressive factors (e.g., cancer‐associated fibroblasts, tumor‐infiltrating lymphocytes, regulatory T cells, myeloid‐derived suppressor cells, tumor‐associated macrophages, and soluble proteins) present in the tumor microenvironment are considered one of the major obstacles for ICD, which supports tumor progression and metastasis and restricts the function of infiltrating antigen‐presenting cells and T cells.^[^ ^19^ ^−^ ^22^ ^]^ Therefore, the development of a novel strategy that can synergistically generate ROS, induce immune response, and inhibit NETs formation is crucial for achieving high‐performance metastatic tumor treatment.

Peptidyl arginine deiminase 4 (PAD4) is an important calcium‐dependent enzyme that can convert arginine from histone 3 to citrulline. The overexpression of PAD4 in a majority of cancers is highly associated with tumor growth and metastasis and plays an important function in NETs formation.^[^ ^23^, ^24^ ^]^ Thus, inhibiting the expression or activity of PAD4 to prevent the formation of NETs is a promising strategy for cancer therapy. It has been reported that PAD4 inhibitors can block NETs formation and perturb natural killer cell and cytotoxic T cells immune response, including taxol, streptomycin, minocycline, GSK199, GSK484, F‐amidine, Cl‐amidine and its derivatives YW3‐56.^[^ ^25^ ^−^ ^28^ ^]^ Among them, YW3‐56 with high potency has been widely used to suppress the citrullination of citrullinated histone H3 (H3cit) and activate a cohort of p53 (tumor suppressor protein)‐target genes, thereby interfering with autophagy and inhibiting cancer cell proliferation.^[^ ^28^ ^−^ ^30^ ^]^ Nevertheless, YW3‐56 usually suffers from poor water solubility, rapid metabolism, and inherent biological toxicity, restricting its clinical development. Combining YW3‐56 with nanomaterials with sonodynamic activity is expected to achieve high‐performance metastatic tumor treatment through US‐triggered ROS production and the resulting ROS‐induced immune responses as well as inhibition of NETs formation.

**Scheme 1:**
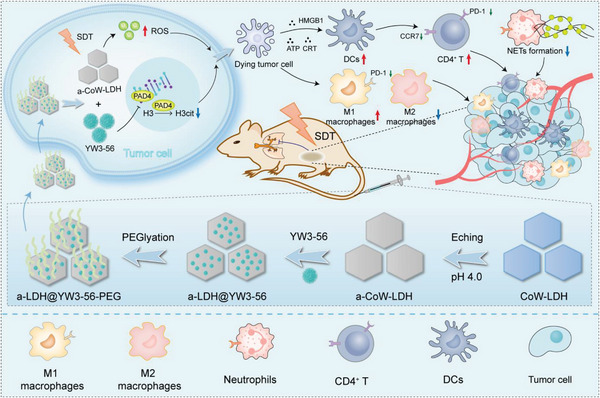
Schematic illustration of the preparation of a‐LDH@356‐PEG nanosheets and their application in synergistic SDT/immunotherapy.

Layered double hydroxide (LDH) nanosheets, as a type of 2D nanomaterials, have been proven to be promising nanoagents in biomedical fields such as tumor diagnosis, cancer therapy, anti‐bacteria, and tissue engineering, by virtue of their pH‐sensitive biodegradability, good biocompatibility, adjustable chemical compositions and structures.^[^ ^31^ ^−^ ^39^ ^]^ In our previous work, the amorphous CoW‐LDH (a‐CoW‐LDH) nanosheets have been demonstrated to function as a sonosensitizer for SDT.^[^ ^15^ ^]^ Herein, we employ the a‐CoW‐LDH nanosheets to load YW3‐56 to skillfully construct a multifunctional nanoagent (a‐LDH@356) for synergistic SDT/immunotherapy (**Scheme** **1**). Specifically, a‐CoW‐LDH nanosheets exhibit superior activity toward ROS generation under US irradiation, including superoxide radicals (·O_2_^−^) and singlet oxygen (^1^O_2_). Importantly, after functionalizing with YW3‐56, a‐LDH@356 plus US irradiation effectively induces ROS generation and ICD, inhibits the elevation of H3cit and the release of NETs, synergistically promoting the anti‐tumor metastasis effect with the least number of lung metastatic nodules. More importantly, in vivo assays demonstrate that the a‐LDH@356 nanosheets can significantly upregulate the proportion of immune‐activated cells, induce polarization of M1 macrophages, and inhibit immune escape by decreasing the expression of PD‐1 on immune cells under US irradiation, thereby suppressing tumor growth with a tumor inhibition rate of 69.5%, which is 1.96‐fold that of YW3‐56 and 1.44‐fold that of a‐LDH, respectively. Our study provides a successful paradigm for the development of a new multifunctional nanoagent for SDT/immunotherapy‐mediated metastatic tumor treatment, demonstrating promising clinical prospects.

## 2 Results and Discussion

### 2.1 Synthesis and Characterization

**Figure 1:**
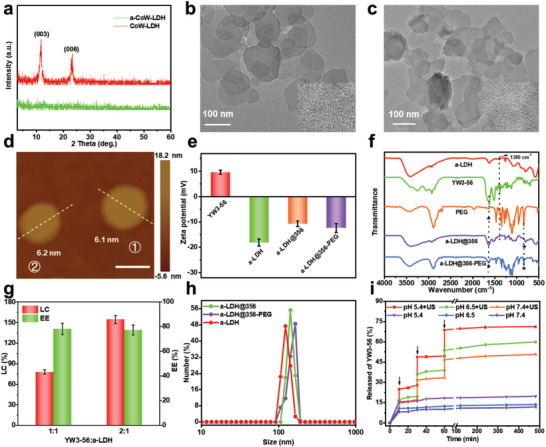
a) XRD patterns of the CoW‐LDH and a‐CoW‐LDH nanosheets. TEM images of b) a‐CoW‐LDH and c) a‐LDH@356 nanosheets. d) AFM image of a‐LDH@356 nanosheets. e) Zeta potentials of YW3‐56, a‐CoW‐LDH, a‐LDH@356, and a‐LDH@356‐PEG nanosheets. f) FT‐IR spectra of YW3‐56, a‐CoW‐LDH, PEG, a‐LDH@356, and a‐LDH@356‐PEG nanosheets. g) LC and EE of YW3‐56 on a‐CoW‐LDH nanosheets with different mass ratios of YW3‐56: a‐CoW‐LDH. h) The hydrodynamic size a‐CoW‐LDH, a‐LDH@356, and a‐LDH@356‐PEG nanosheets. i) YW3‐56 release from a‐LDH@356 at pH 7.4, 6.5, and 5.4 with or without US irradiation (3 W cm^−2^). The arrow indicates the time point of US irradiation.

Pristine CoW‐LDH nanosheets with a size of 80–150 nm were prepared by a hydrothermal method (Figure S1, Supporting Information).^[^ ^15^ ^]^ X‐ray diffraction (XRD) analysis revealed the crystal structure of CoW‐LDH nanosheets, as characteristic diffraction peaks corresponding to the (003) and (006) planes of the LDH crystal were observed (**Figure** **1**a, red line). The lattice fringes with a spacing of 0.38 nm could be ascribed to the (006) planes of LDH crystal, as indicated by high‐resolution transmission electron microscopy (HRTEM, Figure S1, Supporting Information inset). After etching in PBS at pH 4.0 for 6 h, the crystalline CoW‐LDH was transformed into a‐CoW‐LDH with no diffraction peaks found in its XRD pattern (Figure 1a, green line), demonstrating its amorphous phase. The obtained a‐CoW‐LDH nanosheets showed a similar nanosheet morphology to CoW‐LDH with a size of 50–120 nm, while no obvious lattice fringes were found (Figure 1b), further confirming its amorphous structure.

After the loading of YW3‐56, the nanosheet morphology of a‐LDH@356 is well maintained (Figure 1c). The thickness of a‐LDH@356 was measured to be 6.1–6.2 nm (Figure 1d; Figure S2, Supporting Information) according to the atomic force microscopy (AFM). The zeta potentials of a‐LDH nanosheets before and after YW3‐56 (9.6 ± 0.8 mV) loading were −18.1 ± 1.4 and −10.7 ± 1.1 mV (Figure 1e), respectively, suggesting the successful combination of YW3‐56 and a‐CoW‐LDH. The drug loading was further confirmed by UV–vis and Fourier transform infrared (FT‐IR) spectroscopy. In Figure S3 (Supporting Information), the characteristic UV absorption peak of YW3‐56 at 253 nm was observed in the a‐LDH@356 sample. Similarly, the absorption bands of YW3‐56 at 1621 cm^−1^ (C═O stretching vibration in amide bonds) and 1505–1448 cm^−1^ (benzene ring stretching vibration) and a‐LDH at 1380 cm^−1^ (N─O stretching vibration in NO_3_^¯^) were found in the FT‐IR spectrum of a‐LDH@356 nanosheets (Figure 1f), verifying the successful loading of YW3‐56.

The loading performance toward YW3‐56 was then studied by recording the UV absorption at 253 nm of the suspension, and the results showed that the loading content (LC) and encapsulation efficiency (EE) obtained with mass ratio of YW3‐56: a‐LDH = 2:1 were 154.4% and 77.2% (Figure S4, Supporting Information; (Figure 1g), respectively, while the LC and EE of YW3‐56: a‐LDH = 1:1 were 78.1% and 78.1%, respectively. Given that the high concentration of YW3‐56 loaded at YW3‐56: a‐LDH = 2:1 may cause toxic effects, the YW3‐56: a‐LDH = 1:1 sample was selected for subsequent testing. In addition, the hydrodynamic sizes of a‐LDH and a‐LDH@356 are 119.5 ± 3.5 and 127.8 ± 3.7 nm, respectively (Figure 1h). Based on the above results, the release behavior of YW3‐56 from a‐LDH@356 was investigated with external stimuli (pH environment and US irradiation). As shown in Figure 1i, the release amounts of YW3‐56 after incubation at pH 7.4, 6.5, and 5.4 for 8 h were 11.9%, 13.7% and 19.7%, respectively. While 50.7% (pH 7.4), 59.8% (pH 6.5) and 71.2% (pH 5.4) of YW3‐56 was released from a‐LDH@356 under US irradiation (3 W cm^−2^) for 3 min at several time points (10, 30, 60 min), indicating that acid environment and US irradiation can promote the release of YW3‐56 due to the partial dissolution of a‐LDH and cavitation effect of US.

### 2.2 ^1^O_2_‐Generating Activity of a‐LDH@356 Nanosheets

**Figure 2:**
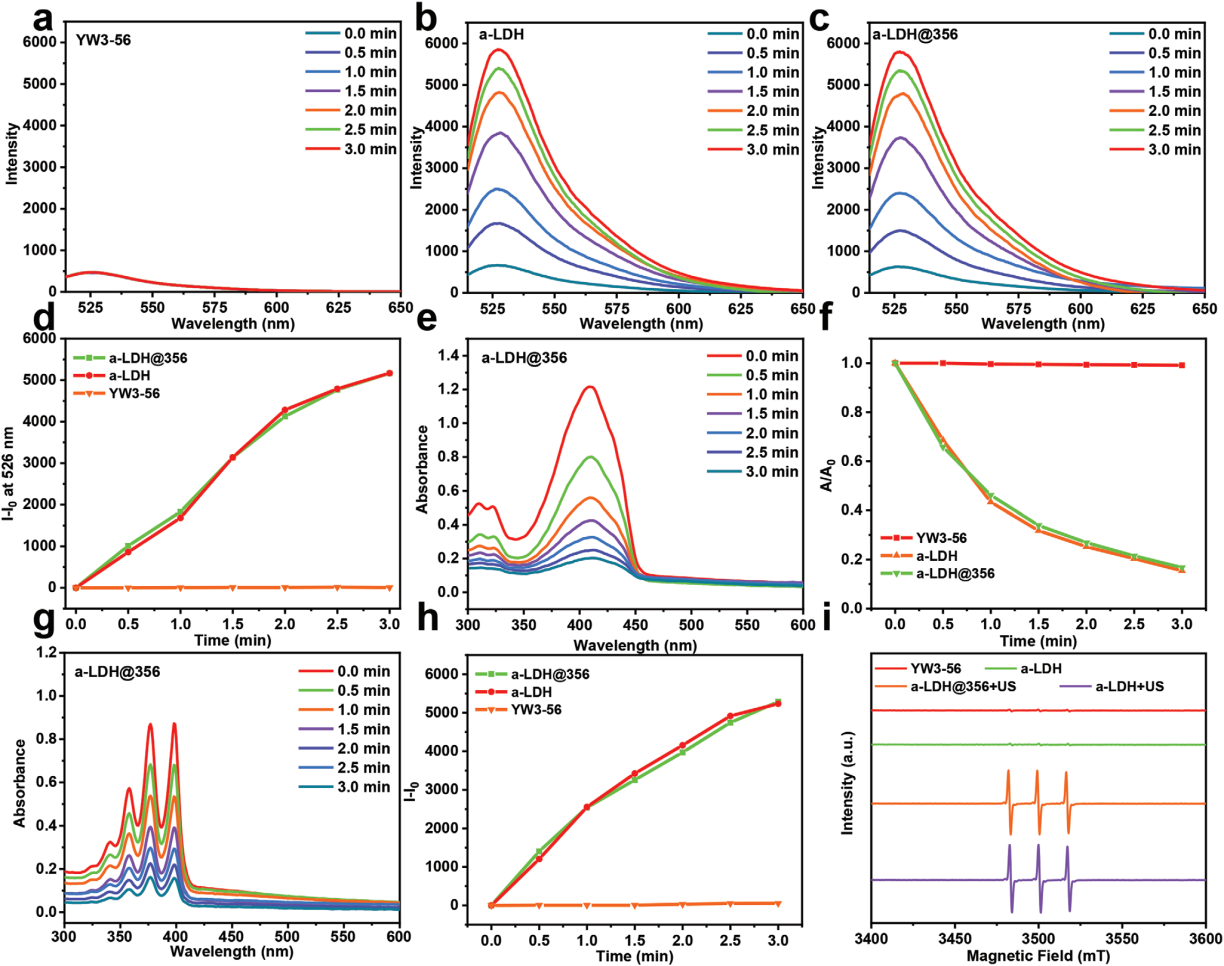
The fluorescence spectra of SOSG (in H_2_O) in the presence of a) pure YW3‐56, b) a‐CoW‐LDH, and c) a‐LDH@356 nanosheets under US irradiation (40 kHz, 3 W cm^−2^). d) The corresponding fluorescence intensity of SOSG (in H_2_O) at 526 nm. e) UV–vis spectra of DPBF (in H_2_O) in the presence of a‐LDH@356 nanosheets under US irradiation (40 kHz, 3 W cm^−2^). f) Normalized absorbance of DPBF (in H_2_O) in the presence of pure YW3‐56, a‐CoW‐LDH and a‐LDH@356 under US irradiation. g) UV–vis spectra of ABDA (in H_2_O) in the presence of a‐LDH@356 nanosheets under US irradiation (40 kHz, 3 W cm^−2^). h) Fluorescence intensity of DCF (in H_2_O) under US irradiation (40 kHz, 3 W cm^−2^) after the addition of pure YW3‐56, a‐CoW‐LDH and a‐LDH@356, respectively. i) ESR spectra of TEMP/^1^O_2_ (in H_2_O) for pure YW3‐56, a‐CoW‐LDH and a‐LDH@356 under US irradiation.

To evaluate the ability of a‐LDH@356 to produce ROS under US irradiation, the singlet oxygen sensor green (SOSG) was selected as the ^1^O_2_ sensing agent.^[^ ^40^, ^41^ ^]^ As presented in **Figure** **2**a–d, the SOSG fluorescence intensity (at 526 nm) in the presence of a‐CoW‐LDH nanosheets increased significantly with the increase of US irradiation time, indicating its excellent ^1^O_2_‐generating activity. In contrast, pure YW3‐56 exhibits no ^1^O_2_‐generating activity. It was found that a‐LDH@356 possesses similar activity to a‐CoW‐LDH for ^1^O_2_ generation, suggesting that the loading of YW3‐56 cannot affect the SDT performance of a‐CoW‐LDH. 1,3‐Diphenylisobenzofuran (DPBF) and 9,10‐anthracenediyl‐bis(methylene) dimalonic acid (ABDA) assays were further utilized to validate the ROS generation performance of a‐LDH@356 nanosheets.^[^ ^42^, ^43^ ^]^ In Figure 2e–g, Figures S5 and S6 (Supporting Information), a negligible decrease in the absorbance of DPBF (0.9%) and ABDA (4.8%) solution containing pure YW3‐56 was observed under US irradiation. Promisingly, the irradiation of a‐CoW‐LDH nanosheets resulted in a significant decrease in the absorbance of DPBF (84.6%) and ABDA (85.1%) solution, while a‐LDH@356 nanosheets induced a similar decrease in the absorbance of DPBF (83.3%) and ABDA (82.4%) solution, demonstrating their satisfactory ROS generation performance. The results were further confirmed by 2′,7′‐dichlorofluorescein diacetate (DCFH‐DA) assay and electron spin resonance (ESR) spectroscopy,^[^ ^44^, ^45^ ^]^ as strong the fluorescence signal of 2′,7′‐dichlorofluorescein (DCF) (Figure 2h; Figure S7, Supporting Information) and characteristic ^1^O_2_ (1:1:1) signal (Figure 2i) were found in the a‐CoW‐LDH and a‐LDH@356 groups.

The mechanism of ROS generation mediated by a‐CoW‐LDH (a‐LDH@356) + US has been elucidated in our previous work.^[^ ^15^ ^]^ Under US irradiation, the electron–hole (e^−^–h^+^) pairs located in the valence band of the a‐CoW‐LDH nanosheets are excited and separated, with h^+^ and e^−^ occupying the valence band and conduction band, respectively. The electrons are ejected to surroundings and react with O_2_ to generate intermediate ·O_2_^−^, which can further combine with h^+^ to form final ^1^O_2_. To confirm the generation of ·O_2_^−^, dihydrorhodamine 123 (DHR 123) assay was carried out. In Figure S8 (Supporting Information), strong fluorescence intensity of DHR123 was found in the presence of a‐CoW‐LDH or a‐LDH@356 under US irradiation, while negligible fluorescence was observed in prue YW3‐56 group, verifying the generation of intermediate ·O_2_^−^.

### 2.3 Modification of a‐LDH@356 Nanosheets with PEG

The aforementioned results have proved the potential of a‐LDH@356 as a high‐efficiency sonosensitizer for SDT. To improve its biosafety, PEG was utilized to modify a‐LDH@356 sample to obtain the a‐LDH@356‐PEG nanosheets. FT‐IR spectroscopy confirmed the successful PEGylation of a‐LDH@356 (Figure 1f), as the absorption band of PEG at 841 cm^−1^ (stretching vibration of C─O─C) was found in a‐LDH@356‐PEG nanosheets. After PEGylation, the zeta potential of a‐LDH@356 nanosheets changed from −10.7 ± 1.1 to −12.4 ± 1.8 mV (Figure 1e). The surface chemical composition of a‐LDH@356‐PEG was investigated by X‐ray photoelectron spectroscopy (XPS). As observed in Figure S9a (Supporting Information), the presence of Co, W, O, C, and N elements was confirmed by the survey spectra of a‐LDH@356‐PEG. As indicated by the high‐resolution XPS Co 2*p* spectra in Figure S9b (Supporting Information), four binding peaks at 797.43, 781.21, 799.62 and 783.18 eV can be assigned to the Co^3+^ 2p_1/2_, Co^3+^ 2p_3/2_, Co^2+^ 2p_1/2_ and Co^2+^ 2p_3/2_, respectively, revealing the coexistence of Co^2+^ and Co^3+^ in a‐LDH@356‐PEG nanosheets. In Figure S9c (Supporting Information), the high‐resolution XPS O 1s spectrum of a‐LDH@356‐PEG nanosheets shows three peaks at 535.03, 533.46, and 531.70 eV, which can be attributed to the adsorbed oxygen, oxygen vacancy, and lattice oxygen, respectively. The XPS W 4f spectrum of a‐LDH@356‐PEG nanosheets proves the presence of W^6+^ species (37.97 eV (4f_5/2_) and 36.24 eV (4f_7/2_)) and W^5+^ species (37.39 eV (4f_5/2_) and 35.26 eV (4f_7/2_)) in the a‐LDH@356‐PEG nanosheets (Figure S9d, Supporting Information), manifesting the coexistence of W^6+^ and W^5+^. In the XPS C 1s spectrum (Figure S9e, Supporting Information), peaks at 288.06 eV (─C═O─ of YW3‐56), 286.11 eV (─C─O─ of PEG), and 284.81 eV (─C═C‐ of YW3‐56 and ─C─C─ of PEG) indicate the presence of YW3‐56 and PEG. Similarly, three peaks at 399.27, 400.01, and 401.21 eV in the XPS N 1s spectrum (Figure S9f, Supporting Information) can be assigned to the pyridinic N, pyrrolic N, and quaternary N, respectively, further demonstrating the existence of YW3‐56. The hydrodynamic size of a‐LDH@356‐PEG nanosheets is 137.6 ± 4.1 nm (Figure 1h), which remains basically unchanged in water, PBS, and DMEM within two weeks (Figure S10, Supporting Information), indicating its excellent dispersion stability. In addition, PEGylation showed a negligible effect on the loading amount of YW3‐56 on a‐CoW‐LDH nanosheets (Figure S11, Supporting Information). The biosafety of a‐LDH@356‐PEG nanosheets was evaluated via hemolysis assay. As shown in Figure S12 (Supporting Information), even at the maximum concentration of 200 µg mL^−1^, the hemolysis rate of a‐LDH@356‐PEG was <2%, indicating excellent biosafety after PEGylation.

### 2.4 Evaluation of In Vitro Therapeutic Effect

The microstructure of tumor cells before and after drug treatment was characterized by TEM. After treatment with a‐LDH@356‐PEG + US, there was significant drug residue in the intracellular phagocytic vesicles (Figure S13a, Supporting Information). Subsequently, to quantify the cellular uptake of drugs, the intracellular Co content was determined by inductively coupled plasma mass spectrometry (ICP‐MS). Compared with control or control + US group, intracellular Co content in a‐LDH‐PEG and a‐LDH@356‐PEG groups were significantly increased regardless of whether exposed to US irradiation (Figure S13b, Supporting Information). Particularly, the intracellular Co content in a‐LDH@356‐PEG group was significantly increased compared with a‐LDH‐PEG group, which was further enhanced after US irradiation, indicating that YW3‐56 and US irradiation were conducive to the cellular uptake of a‐LDH‐PEG. The uptake mechanism could be explained as that a‐LDH@356‐PEG was internalized by cells through clathrin‐mediated endocytosis and then delivered into cytoplasm via endosomal escape.^[^ ^46^ ^−^ ^48^ ^]^

**Figure 3:**
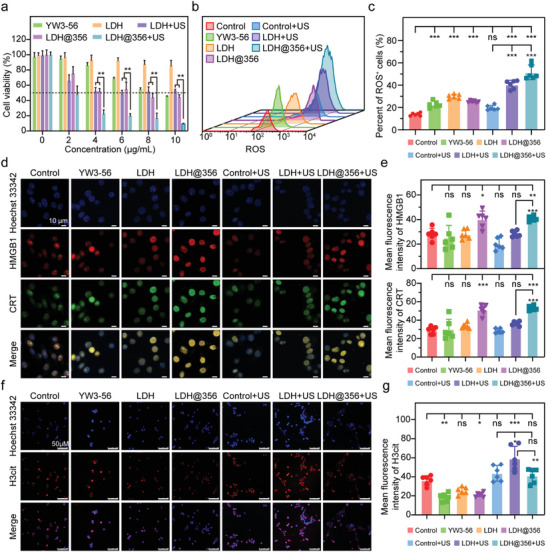
SDT effects of a‐LDH@356‐PEG in vitro. a) Cell viability of 4T1 cells with different treatments. b) Representative results of ROS detected by flow cytometry and c) corresponding quantitative analysis. d) HMGB1 and Calreticulin staining images of 4T1 cells with different treatments and e) corresponding quantitative analysis (Scale bar = 10 µm). f) Influence on ability of NETs releases with different treatments and g) corresponding quantitative analysis (Scale bar = 50 µm). Data are presented as mean ± SD (*n =* 6). Statistical analysis was performed via one‐way ANOVA. *p < 0.05, **p <0.01, ***p < 0.001.

Motivated by the above exciting results, we evaluated the cytotoxicity of YW3‐56, a‐LDH‐PEG, a‐LDH@356‐PEG, a‐LDH‐PEG + US, a‐LDH@356‐PEG + US on 4T1 cells using a standard methyl thiazolyl tetrazolium (MTT) assay (Table S1, Supporting Information). As expected, >90% of cells survived after 24 h of incubation with a‐LDH (0−50 µg mL^−1^), suggesting its preeminent biocompatibility (Figure S14a, Supporting Information). However, the inclusion of YW3‐56 imparted the cytotoxic activity of a‐LDH@356‐PEG to kill 4T1 cells, and the viability of 4T1 cells was decreased to <30% when treated with 4 µg mL^−1^ a‐LDH@356‐PEG under US irradiation (40 kHz, 3W·cm^−2^, 3 min), while that was still >50% when treated with 4 µg mL^−1^ a‐LDH‐PEG + US (**Figure** **3**a). These results suggested that YW3‐56 loading conferred a‐LDH‐PEG the ability of anti‐proliferation in vitro, which was further enhanced by US intervention. Subsequently, wound‐healing assay was carried out to examine the antimetastatic ability of a‐LDH@356‐PEG + US in vitro. As presented in Figure S14b,c (Supporting Information), compared with control or control + US group, the wound‐healing ability of the YW3‐56, a‐LDH‐PEG, a‐LDH@356‐PEG, and a‐LDH‐PEG + US groups was significantly reduced. Moreover, a‐LDH@356‐PEG + US group exhibited further enhanced anti‐wound‐healing ability, indicating its extraordinary antimetastatic ability.

The ROS levels in 4T1 cells were detected after various treatments for 24 h: control (PBS), YW3‐56, a‐LDH‐PEG, a‐LDH@356‐PEG, control + US, a‐LDH‐PEG + US or a‐LDH@356‐PEG + US at the same YW3‐56 equivalent concentration of 2.8 µg mL^−1^. As shown in Figure 3b,c, the ROS levels of a‐LDH‐PEG + US and a‐LDH@356‐PEG + US groups were much higher than that of other groups, demonstrating the excellent ROS generation performance of a‐LDH‐PEG and a‐LDH@356‐PEG. The ability to induce apoptosis was evaluated on 4T1 cells using an Annexin V‐FITC/PI staining probe. As shown in Figure S15 (Supporting Information), after treatment with a‐LDH@356‐PEG + US, the proportion of apoptotic cells was significantly increased compared with that of control + US and a‐LDH‐PEG + US groups, suggesting that a‐LDH@356‐PEG + US could induce apoptosis of tumor cells, which may correspond to an increase in ROS after treatment. To reveal the capacity of a‐LDH@356‐PEG to generate immunogenicity in dying tumor cells and induce ICD, we measured two ICD biomarkers, high‐mobility group box 1 protein (HMGB1) and calreticulin (CRT).^[^ ^49^, ^50^ ^]^ Compared with control group, both HMGB1 and CRT levels in 4T1 cells were significantly enhanced after treatment with a‐LDH@356‐PEG or a‐LDH@356‐PEG + US, which was not observed in a‐LDH‐PEG + US group (Figure 3d,e), proving the activity of YW3‐56 to induce ICD. Besides, CRT content determined by western blot (Figure S16a, Supporting Information) and HMGB1 detected using ELISA (Figure S16b, Supporting Information) were consistent with the CLSM results, which further indicated the ability of a‐LDH@356‐PEG + US to induce ICD. Encouraged by the above results, another ICD biomarker adenosine triphosphate (ATP) was also detected. Interestingly, compared with control group, the endogenous ATP level was significantly reduced in both a‐LDH‐PEG and a‐LDH‐PEG + US group (Figure S14d, Supporting Information), which might be attributed to the inhibition of H^+^‐ATP synthase activity by a‐LDH‐PEG‐induced ROS. Moreover, a‐LDH@356‐PEG + US group had a significantly higher ATP level than control + US and a‐LDH@356‐PEG group, suggesting the synergistic effect of YW3‐56 and US‐triggered ROS generation (Figure S14d, Supporting Information).

To observe the effect of a‐LDH@356‐PEG + US on NETs release from neutrophils, mouse neutrophils were stimulated with 5 µmol L^−1^ A23187 for 2 h.^[^ ^24^, ^51^ ^]^ As shown in control and control + US groups, the extracellular fibers with co‐localization of the highly decondensed DNA (blue) and H3cit (red) are called NETs (Figure 3f). The mean fluorescence intensity of H3cit in YW3‐56 and a‐LDH@356‐PEG groups was lower than that in control group, suggesting the regression of overall H3cit level (Figure 3f,g). Notably, overall H3cit level and NETs release increased in a‐LDH‐PEG + US group compared with control group, which was effectively attenuated in a‐LDH@356‐PEG + US group, indicating that a‐LDH@356‐PEG could effectively inhibit the elevation of H3cit and the release of NETs under US irradiation. Similarly, treatment with a‐LDH@356‐PEG + US in 4T1 cells reversed the overall increase in H3cit induced by a‐LDH‐PEG + US (Figure S17a,b, Supporting Information).

### 2.5 Evaluation of In Vivo Therapeutic Effect

**Figure 4:**
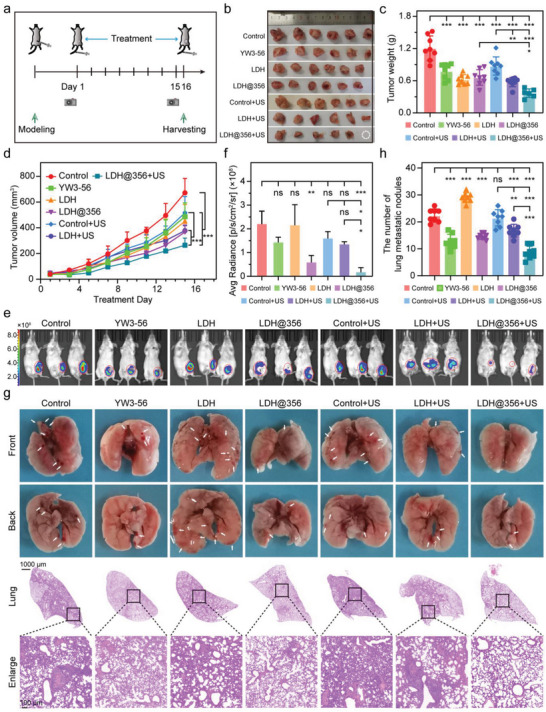
Antitumor and anti‐metastasis properties of a‐LDH@356‐PEG in vivo. a) Treatment scheme of orthotopic 4T1 tumor‐bearing mice. Control (normal saline), YW3‐56 (0.35 mg mL^−1^), a‐LDH‐PEG (0.5 mg mL^−1^), a‐LDH@356‐PEG (0.5 mg mL^−1^), control + US, a‐LDH‐PEG + US (0.5 mg mL^−1^), and a‐LDH@356‐PEG + US (0.5 mg mL^−1^) were intravenously injected every two days, respectively. b) Representative photos and c) weights of the harvested tumors. d) Tumor volume curves of different groups of mice recorded every two days. e) Living images of orthotopic 4T1 tumor‐bearing mice on 15th day after various treatments and f) corresponding quantitative analysis. g) Representative images of metastasis in lung tissues and H&E staining of lung sections and h) corresponding quantitative analysis. Data are presented as mean ± SD (*n =* 6). Statistical analysis was performed via one‐way ANOVA. *p < 0.05, **p <0.01, ***p < 0.001.

Inspired by the above in vitro results, an orthotopic 4T1 tumor‐bearing mice model was utilized to evaluate the antitumor and anti‐metastasis activities of a‐LDH@356‐PEG in vivo. Each treatment regimen was administered every two days until the tumor was harvested on 16th day, and the living images were acquired on the 1st and 15th day of treatment (**Figure** **4**a). As shown in Figure 4b,c, each treatment inhibited tumor growth to varying degrees. Interestingly, the tumor weight of a‐LDH@356‐PEG group was significantly lower than that of control group, which was further decreased plus US irradiation with a tumor inhibition rate of 69.5%. The tumor volume curve also revealed that a‐LDH@356‐PEG + US group had the most prominent ability to inhibit tumor growth in situ (Figure 4d). The above conclusion was confirmed again by the acquisition of living images of the mice on the 1^st^ and 15th day of administration (Figure 4e,f; Figure S18a,b, Supporting Information), as a‐LDH@356‐PEG + US group exhibited the weakest bioluminescence at tumor site on 15th day. Moreover, the photographs and H&E staining of the lung tissues showed that the tissues from a‐LDH@356‐PEG + US group had the least number of lung metastatic nodules and normal structures without histopathologic changes, suggesting its most outstanding anti‐metastasis ability (Figure 4g,h).

**Figure 5:**
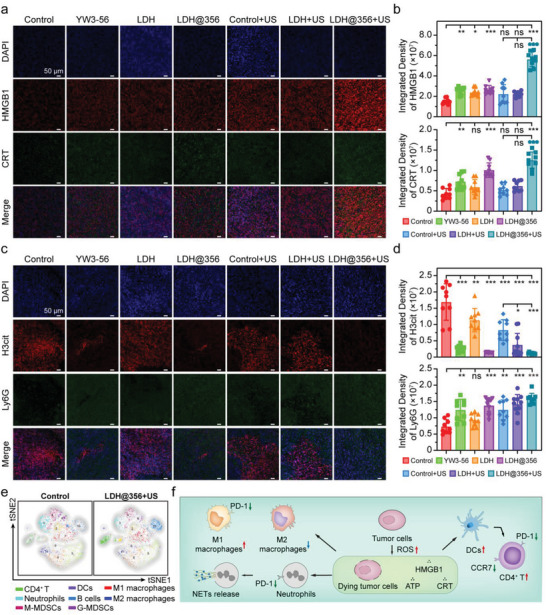
Immunogenic cell death and NETs release in the tumor microenvironment. a) The immunohistochemistry staining of HMGB1 and CRT in tumor sections and b) corresponding quantitative analysis. c) The immunohistochemistry staining of H3cit and Ly6G in tumor sections and d) corresponding quantitative analysis. e) tSNE diagram of immune cells in tumor tissues determined by single‐cell flow mass cytometry. f) ICD process induced by a‐LDH@356‐PEG + US. Data are presented as mean ± SD (*n =* 6). Statistical analysis was performed via one‐way ANOVA. *p < 0.05, **p < 0.01, ***p < 0.001.

To assess the biocompatibility in vivo, the body weights of mice in each group during administration and the viscerosomatic ratio of major organs after different treatments were recorded. No significant changes in body weights and visceral‐body ratio were observed between the a‐LDH@356‐PEG + US and control + US groups (Figure S19a–f, Supporting Information). The H&E staining images of major organ sections also showed that there were no apparent physiological morphology abnormalities in heart, liver, spleen, and kidney treated with a‐LDH@356‐PEG + US (Figure S20, Supporting Information). Collectively, these results fully demonstrated the negligible biotoxicity of a‐LDH@356‐PEG + US, strengthening its further application for synergetic SDT/immunotherapy. We further adopted an immunohistochemistry staining assay to evaluate the ICD in the tumor microenvironment. The a‐LDH@356‐PEG + US group showed the strongest HMGB1 and CRT fluorescence, indicating enhanced immunogenic tumor cell death (**Figure** **5**a,b). Moreover, the a‐LDH@356‐PEG + US group showed the weakest H3cit fluorescence and the strongest Ly6G fluorescence, confirming the inhibited NETs release and the increased neutrophil infiltration in tumor (Figure 5c,d).

To elucidate the specific mechanism of a‐LDH@356‐PEG inhibiting the formation of SDT‐mediated NETs, several proteins associated with NETs were detected by immunohistochemistry. It has been reported that SDT may cause the increase of inflammatory factor IL‐1β while killing tumor cells to induce ICD,^[^ ^10^ ^]^ thus promoting the formation of NETs and inducing sustained inflammatory reaction.^[^ ^52^ ^]^ Therefore, IL‐1β may be the link hub between ICD and NETs. In view of this, the IL‐1β levels in tumor tissue were detected after treatment with a‐LDH‐PEG + US and a‐LDH@356‐PEG + US. As shown in Figure S21 (Supporting Information), an increase in IL‐1β level was observed in a‐LDH@356‐PEG + US group compared with a‐LDH‐PEG + US group. The increase of IL‐1β would theoretically lead to an increase in NETs. However, it was observed that a‐LDH@356‐PEG + US produced fewer NETs than a‐LDH‐PEG + US (Figure 5c,d), suggesting that YW3‐56 could effectively inhibit SDT‐mediated NETs formation through IL‐1β. In addition, it has also been reported that NETs could dissolve the extracellular matrix through its protease MMP9 and MMP2 to improve the ability of tumor invasion.^[^ ^53^ ^]^ Based on this, the MMP9 and MMP2 levels in tumor tissues were evaluated after treatment with a‐LDH@356‐PEG + US. Histological results showed the decreased expression of MMP9 and MMP2 in tumor tissues after administrated with a‐LDH@356‐PEG + US (Figure S21, Supporting Information), revealing that YW3‐56 could inhibit tumor metastasis through the NETs‐MMP9‐MMP2 pathway.

Subsequently, we prepared single‐cell suspensions of tumor tissues from 4T1 tumor‐bearing mice for flow mass cytometry.^[^ ^54^, ^55^ ^]^ Cells were grouped according to the different specific antibody expressions of immune cells (Figure 5e), and their proportions were measured (Figure S22a, Supporting Information). The proportions of CD4^+^ T cells and DCs in a‐LDH@356‐PEG + US group were significantly higher than that in control group, indicating the activation of innate immunity by a‐LDH@356‐PEG + US. Moreover, the proportion of M1 macrophage cells increased and the proportion of M2 macrophage cells decreased, suggesting that a‐LDH@356‐PEG + US induced antitumor phenotype by promoting M1 macrophage polarization (Figure S22a, Supporting Information). The upregulation of CCR7 for CD4^+^ T cells in a‐LDH@356‐PEG + US group induced T cells to selectively target tissues and initiated autoimmune responses (Figure S22b, Supporting Information). The down‐regulation of PD‐1 for immune cells (e.g., CD4^+^ T cells, neutrophil, M‐MDSCs, G‐MDSCs, M1 macrophages cells) in a‐LDH@356‐PEG + US group played a great role in inhibiting immune escape (Figure S22c, Supporting Information). Overall, a‐LDH@356‐PEG + US significantly upregulated the proportion of immune‐activated cells, induced polarization of M1 macrophages, and inhibited immune escape by decreasing the expression of PD‐1 on immune cells (Figure 5f).

## 3 Conclusion

In summary, the a‐CoW‐LDH nanosheets loaded with YW3‐56 (a‐LDH@356) can function as a multifunctional nanoagent for synergistic SDT/immunotherapy. Specifically, a‐CoW‐LDH nanosheets could induce a large number of ROS generation and prominent ICD under US irradiation. Moreover, after loading with YW3‐56, a‐LDH@356‐PEG effectively inhibited the elevation of H3cit and the release of NETs, as evidenced by in vitro assay. US‐triggered ROS production and the resulting ROS‐induced immune responses as well as inhibition of NETs formation synergistically promoted the anti‐tumor metastasis effect of a‐LDH@356‐PEG with the least number of lung metastatic nodules. In vivo assay further demonstrated that a‐LDH@356‐PEG significantly upregulated the proportion of immune‐activated cells, induced polarization of M1 macrophages, and inhibited immune escape by downregulating the expression of PD‐1 on immune cells under US irradiation, thereby suppressing tumor growth with a tumor inhibition rate of 69.5%, which was 1.96‐fold that of YW3‐56 and 1.44‐fold that of a‐LDH‐PEG, respectively, showing promising clinical prospect in the treatment of metastatic tumors. Such a combined tumor‐therapeutic strategy based on sonosensitizer‐mediated SDT and PAD4 inhibitor‐augmented immunotherapy offers a potent immune memory function, protecting against tumor rechallenge after eliminating the primary tumors.

This work has successfully proposed a new strategy for synergistic generation of ROS, induction of immune response, and inhibition of NETs formation, achieving high‐performance metastatic tumor treatment. However, current research mainly focuses on the pursuit of high therapeutic efficiency, neglecting the comprehensive evaluation of biosafety, such as the degradation mechanism and metabolic pathways of a‐LDH@356‐PEG in vivo, which is crucial to ensuring its long‐term biosafety. Although it has been demonstrated that a‐CoW‐LDH‐PEG with good biocompatibility could be metabolized and excreted through the liver and kidney, as detected in feces and urine,^[^ ^15^ ^]^ the long‐term side effects of a‐LDH@356‐PEG are not fully understood, and whether the interaction between a‐LDH and YW3‐56 will cause implicit toxicity is not yet known. Therefore, multiple evaluation techniques and toxicological parameters, as well as underlying mechanism issues should also be considered in future development. In addition, how to effectively avoid the slight shedding of YW3‐56 after intravenous injection of a‐LDH@356‐PEG is also a problem worthy of our in‐depth consideration.

## Conflict of Interest

The authors declare no conflict of interest.

## Supporting information

Supporting Information

## Data Availability

The data that support the findings of this study are available from the corresponding author upon reasonable request.
