# Supplementary material for: PAD4 Inhibitor‐Functionalized Layered Double Hydroxide Nanosheets for Synergistic Sonodynamic Therapy/Immunotherapy Of Tumor Metastasis
Source: Adv Sci (Weinh). 2024 May 6;11(26):2401064. doi: 10.1002/advs.202401064 (PMC11234469; doi:10.1002/advs.202401064)
Supplement: Supplementary file 1 — Supporting Information [file ADVS-11-2401064-s001.docx]

***Supporting information***

PAD4 inhibitor-functionalized layered double hydroxide nanosheets for synergistic sonodynamic therapy/immunotherapy of tumor metastasis

Di Zhu,^†^ Yu Lu,^†^ Shuqing Yang,^†^ Tingting Hu, Chaoliang Tan,* Ruizheng Liang,* and Yuji Wang*

^†^These authors contributed equally: Di Zhu, Yu Lu, Shuqing Yang.

D. Zhu, Dr. Y. Lu and Prof. Y. Wang

Department of Medicinal Chemistry, College of Pharmaceutical Sciences of Capital Medical University, Beijing, 100069, P. R. China

E-mail: [wangyuji@ccmu.edu.cn](mailto:wangyuji@ccmu.edu.cn)

Prof. Y. Wang

Beijing Area Major Laboratory of Peptide and Small Molecular Drugs, Engineering Research Center of Endogenous Prophylactic of Ministry of Education of China, Beijing Laboratory of Biomedical Materials, Laboratory for Clinical Medicine, Capital Medical University, Beijing Laboratory of Oral Health, Beijing 100069, P. R. China

S. Yang and Prof. R. Liang

State Key Laboratory of Chemical Resource Engineering, Beijing Advanced Innovation Center for Soft Matter Science and Engineering, Beijing University of Chemical Technology, Beijing 100029, P. R. China

E-mail: [liangrz@mail.buct.edu.cn](mailto:liangrz@mail.buct.edu.cn)

Prof. R. Liang

Quzhou Institute for Innovation in Resource Chemical Engineering, Quzhou 324000, P. R. China

Dr. T. Hu and Prof. C. Tan

Department Electrical and Electronic Engineering, The University of Hong Kong, Pokfulam Road, Hong Kong SAR 999077, P. R. China

E-mail: [cltan@hku.hk](mailto:cltan@hku.hk)

**Experimental Section**

**1. Materials and reagents**

Cobalt nitrate hexahydrate (Co(NO_3_)_2_·6H_2_O, >99.0%), sodium tungstate dihydrate (Na_2_WO_4_·2H_2_O, 99.5%), sodium hydroxide (NaOH, >98.0%), nitric acid (HNO_3_), 9,10-anthracenediyl-bis(methylene) dimalonic acid (ABDA), singlet oxygen sensor green (SOSG), 1,3-diphenylisobenzonfuran (DPBF), 2,2,6,6-tetramethyl-4-piperidone (TEMP), and methyl thiazolyl tetrazolium (MTT) were acquired from Aladdin Reagent (Shanghai, China). Dimethyl sulfoxide (DMSO) and 2’,7’-dichlorofluorescein diacetate (DCFH-DA) were obtained from Sigma-Aldrich and Fisher (USA). PBS (KGB5001), RPMI-1640 medium (KGM31800), 0.25% trypsin-EDTA digestive fluid (KGY0012) were purchased from KeyGEN BioTECH (Jiangsu, China). PBST (P1031), 4% polyformaldehyde (P1110), 5%BSA blocking solution (SW3015) were purchased from Solarbio (Beijing, China). anti-fluorescence quenching sequester (P0126), DAPI (C1006), Hoechst 33342 (C1026), reactive oxygen species detection kit (S0033M), and mouse adenosine triphosphate (ATP) elisa kit (S0026) were purchased from Beyotime (Jiangsu, China). Anti-histone H3 (citrulline R2+R8+R17) antibody (ab5103), rabbit monoclonal [EPR3507] to HMGB1 (ab79823), alexa fluor®488 rabbit monoclonal [EPR3924] to Calreticulin-ER marker (ab196158), and goat anti-rabbit IgG H&L (alexa fluor®568) (ab175471) were obtained from Abcam (English). FITC-labeled anti-mouse Ly6g monoclonal antibody (11-9668-82) was obtained from ThermoFisher Scientific (US).

4T1 (mouse breast carcinoma cells), 4T1-Luc (mouse breast carcinoma cells transduction with luciferase gene) were purchased from KeyGEN BioTECH. The experimental animal BALB/c female mice were bought from Beijing Vital River Laboratory Animal Technology Co., Ltd.

**2. Characterizations**

Powder X-ray diffraction (XRD) patterns of the sample were acquired from a Shimadzu XRD-6000 diffractometer (Cu Kα radiation source, λ= 0.15406 nm). Transmission electron microscopy (TEM) images were taken from a JEOL transmission electron microscope (JEM-2010, accelerating voltage of 200 kV, Tokyo, Japan). An atomic force microscope (AFM, MultiMode 8, Bruker) was utilized to detect the thickness of the samples in tapping mode. Bruker EMX1598 spectrometer was used to measure the electron spin resonance (ESR) spectra of the samples. A Zetasizer UV spectrometer (Malvern Instruments, U.K.) was adopted to investigate the hydrodynamic sizes and zeta potentials of the samples.

**3. Methods**

**3.1 Preparation of CoW-LDH and a-CoW-LDH nanosheets**

CoW-LDH nanosheets were prepared using hydrothermal method according to literature reports. Firstly, Na_2_WO_4_·2H_2_O (0.5 mmol) was added to 30 mL deionized water and mixed with HNO_3_ (0.4 mL) to make the full dissolution of Na_2_WO_4_·2H_2_O under ultrasonic treatment. Then, Co(NO_3_)_2_·6H_2_O (0.5 mmol) dissolved in 20 mL deionized water was slowly dropped into the above solution under N_2_ protection and magnetically stirred for 10 min. Subsequently, NaOH (0.05 mol) was dissolved in 50 mL deionized water and then added to the obtained mixed solution at 80 °C with pH = 9.70. After magnetic stirring for 90 min, the resulting precipitate was transferred to a Teflon autoclave reactor (100 mL) and heated at 80 °C for 24 h. Finally, the resultant CoW-LDH nanosheets were washed with deionized water *via* centrifugation. The a-CoW-LDH nanosheets were prepared by etching CoW-LDH nanosheets in buffer solution at pH 4.0 for 6 h.

**3.2 Preparation of a-LDH@356 and a-LDH@356-PEG nanosheets**

YW3-56 (20 mg) was dissolved in PBS (10 mL) and then added to a-CoW-LDH suspension (10 mg dispersed in 10 mL PBS). The mixture was magnetically stirred at room temperature for 24 h and centrifuged at 6000 rpm to obtain a-LDH@356. To prepare a-LDH@356-PEG sample, PEG (100 mg) was added to a-LDH@356 suspension (10 mg dispersed in 10 mL PBS) under ultrasonication for 0.5 h and then magnetically stirred at room temperature for 8 h. The resulting a-LDH@356-PEG was washed with deionized water for four times and then re-dispersed in deionized water for further use.

**3.3 Evaluation of singlet oxygen (^1^O_2_) generation**

The ^1^O_2_ generation performance of YW3-56, a-CoW-LDH and a-LDH@356 was evaluated by SOSG, DCFH-DA, DPBF, ABDA, and ESR assays. For SOSG assay, YW3-56 (0.2 mL, 0.78 mg mL^−1^), a-CoW-LDH (0.2 mL, 1 mg mL^−1^) and a-LDH@356 (0.2 mL, 1 mg mL^−1^ of a-LDH) were added to 1.8 mL H_2_O containing SOSG (2 μM), respectively. Then, these obtained mixtures were irradiated by US (40 kHz, 3 W cm^−2^) for 3 min. The generated ^1^O_2_ was indirectly detected by recording the fluorescence spectra of SOSG every 30 s. For DCFH-DA assay, the generated ^1^O_2_ was detected by similar procedures except that SOSG (2 μM) was changed to DCFH-DA (6 mM).

In terms of DPBF assay, YW3-56 (80 μL, 1.95 mg mL^−1^), a-CoW-LDH (80 μL, 2.5 mg mL^−1^) and a-LDH@356 (80 μL, 2.5 mg mL^−1^ of a-LDH) were added to 1920 μL H_2_O containing DPBF (25 μg mL^−1^), respectively. The obtained mixtures were exposed to US irradiation (40 kHz, 3 W cm^−2^) for 3 min. The ^1^O_2_ generation was indirectly detected by recording the absorbance of DPBF at 300~600 nm every 30 s. For ABDA assay, the generated ^1^O_2_ was detected by similar procedures except that DPBF (25 μg mL^−1^) was changed to ABDA (0.2 mM).

As for ESR assay, TEMP was used as a trapping probe to verify the ^1^O_2_ generation. Briefly, TEMP (0.1 mL, 90 mM) was mixed with the YW3-56 (0.1 mL, 0.156 mg mL^−1^), a-CoW-LDH (0.1 mL, 0.2 mg mL^−1^) and a-LDH@356 solution (0.1 mL, 0.2 mg mL^−1^), respectively. Then, the generated ^1^O_2_ was detected by ESR spectrometer after US irradiation (40 kHz, 3 W cm^−2^) for 3 min.

**3.4** ***In vitro* cytotoxicity**

MTT assay was used to determine the anti-tumor cell proliferation *in vitro*. 4T1 cells were seeded in a 96-well plate (3×10^3^ cells/well) in 100 μL of RPMI-1640 (10% FBS) and cultured overnight. Then, 25 μL of RPMI-1640 containing YW3-56, a-LDH-PEG, a-LDH@356-PEG, a-LDH-PEG + US and a-LDH@356-PEG + US at different concentrations were added respectively and co-incubated for 24 h. As for the a-LDH-PEG + US and a-LDH@356-PEG + US group, 4T1 cells were exposed to US irradiation (40 kHz, 3W·cm^−2^) at the 4^th^ h for 3 min. MTT assay was executed according to the traditional protocol, while data were analyzed by Graphpad Prism 9.5.2.

**3.5** ***In vitro* ROS generation**

For measuring the ROS generation, 4T1 cells were seeded in a 6-well plate (2×10^5^ cells/well) in 2 mL of RPMI-1640 (10% FBS) and cultured overnight. Then, they were incubated with control (PBS), YW3-56 (2.8 μg mL^−1^), a-LDH-PEG (4 μg mL^−1^), a-LDH@356-PEG (4 μg mL^−1^), control + US, a-LDH-PEG + US (4 μg mL^−1^), and a-LDH@356-PEG + US (4 μg mL^−1^) for 24 h, respectively. US irradiation (40 kHz, 3W·cm^−2^) was performed at the 4^th^ h for 3 min. After rinsing with PBS, the cells were labeled with DCFH-DA and subsequently detected by flow cytometry (BD LSRFortessa SORP, USA).

**3.6** **Assessment of extracellular ATP level**

To measure the extracellular adenosine triphosphate (ATP) level, 4T1 cells were seeded in a 6-well plate (2×10^5^ cells/well) and cultured overnight, followed by incubation with control (PBS), YW3-56 (2.8 μg mL^−1^), a-LDH-PEG (4 μg mL^−1^), a-LDH@356-PEG (4 μg mL^−1^), control + US, a-LDH-PEG + US (4 μg mL^−1^), and a-LDH@356-PEG + US (4 μg mL^−1^) for 24 h, respectively. US irradiation (40 kHz, 3W·cm^−2^) was performed at the 4^th^ h for 3 min. After rinsing with PBS, the cells were lysed to obtain the supernatant. Subsequently, the intracellular ATP level was measured using a corresponding testing kit *via* SpectraMax®iD5 (Molecular Devices, USA).

**3.7** **Wound-healing assay**

For evaluating wound-healing ability, 4T1 cells were seeded in a 6-well plate (2×10^5^ cells/well) and cultured for 24 h. The cell surface was scratch-wounded using sterilized pipette and rinsed with PBS, followed by incubation with control (PBS), YW3-56 (2.8 μg mL^−1^), a-LDH-PEG (4 μg mL^−1^), a-LDH@356-PEG (4 μg mL^−1^), control + US, a-LDH-PEG + US (4 μg/mL), and a-LDH@356-PEG + US (4 μg mL^−1^) for 48 h, respectively. US irradiation (40 kHz, 3W·cm^−2^) was performed at the 4^th^ h for 3 min. Then, the plates were photographed *via* Zen Blue 3.1 Imaging System.

**3.8** **Expression of HMGB1 and calreticulin**

4T1 cells (1×10^5^ cells/well) were seeded in a confocal dish and cultured overnight, followed by incubation with control (PBS), YW3-56 (2.8 μg mL^−1^), a-LDH-PEG (4 μg mL^−1^), a-LDH@356-PEG (4 μg mL^−1^), control + US, a-LDH-PEG + US (4 μg mL^−1^), and a-LDH@356-PEG + US (4 μg mL^−1^) for 24 h, respectively. US irradiation (40 kHz, 3W·cm^−2^) was performed at the 4^th^ h for 3 min. After removing the medium, the cells were fixed with 4% polyformaldehyde (containing 0.2% Triton X-100) for 15 min at 4 °C and blocked with 5% BSA for 1 h at 25 °C. The cells were then treataed with anti-HMGB1 antibody (abcam, ab79823) overnight at 4 °C and Goat Anti-Rabbit IgG H&L (Alexa Fluor®568) (abcam, ab175471) for 1 h at 25 °C, followed by Alexa Fluor®488 Rabbit monoclonal to Calreticulin (abcam, ab196158) for 1 h at 25 °C. Nuclear DNA was stained with Hoechst 33342 for 3 min and washed with PBS. Finally, the mean fluorescence intensity was measured by a laser confocal microscope (TCS SP8 STED, Leica, Germany).

**3.9** **Assessments of H3cit level**

4T1 cells (1×10^5^ cells/well) were seeded in a confocal dish and cultured overnight, followed by incubation with control (PBS), YW3-56 (2.8 μg mL^−1^), a-LDH-PEG (4 μg mL^−1^), a-LDH@356-PEG (4 μg mL^−1^), control + US, a-LDH-PEG + US (4 μg mL^−1^), and a-LDH@356-PEG + US (4 μg mL^−1^) for 24 h, respectively. US irradiation (40 kHz, 3W·cm^−2^) was performed at the 4^th^ h for 3 min. After removing the medium, the cells were fixed with 4% polyformaldehyde (containing 0.2% Triton X-100) for 15 min at 4 °C and blocked with 5% BSA for 1 h at 25 °C. The cells were then incubated with anti-Histone H3 (citrulline R2+R8+R17) antibody (abcam, ab5103) overnight at 4 °C, and Goat Anti-Rabbit IgG H&L (Alexa Fluor® 568) (abcam, ab175471) for 1 h at 25 °C. Nuclear DNA was stained with Hoechst 33342 for 3 min and washed with PBS. Finally, the mean fluorescence intensity was measured by a laser confocal microscope (TCS SP8 STED, Leica, Germany).

**3.10** **NETs formation assay**

After sacrifice of BALB/C mice by cervical dislocation, the bone marrow was isolated from femurs and tibia in a sterile manner, and then suspended by PBS and filtered by 70 μm nylon filter membrane to obtain single-cell suspension. Neutrophils were isolated using a mouse neutrophil isolation kit (TBDSceicge, LZS1100), and seeded in a confocal dish at a density of 5×10^5^ cells per well, followed by incubation with control (PBS), YW3-56 (2.8 μg mL^−1^), a-LDH-PEG (4 μg mL^−1^), a-LDH@356-PEG (4 μg mL^−1^), control + US, a-LDH-PEG + US (4 μg mL^−1^), and a-LDH@356-PEG + US (4 μg mL^−1^) for 2 h, respectively. US irradiation (40 kHz, 3W·cm^−2^) was performed at the first h for 3 min. Then, calcium ionophore A231875 (5 μmol/L) was added and incubated for another 2 h to induce NETosis. After centrifugation to remove the medium, the cells were fixed with 4% polyformaldehyde (containing 0.2% Triton X-100) for 15 min at 4 °C and blocked with 5% BSA for 1 h at 25 °C. The cells were then incubated with anti-Histone H3 (citrulline R2+R8+R17) antibody (abcam, ab5103) overnight at 4 °C, and Goat Anti-Rabbit IgG H&L (Alexa Fluor® 568) (abcam, ab175471) for 1 h at 25 °C. Nuclear DNA was stained with Hoechst 33342 for 3 min and washed with PBS. Finally, the mean fluorescence intensity was measured by a laser confocal microscope (TCS SP8 STED, Leica, Germany).

**3.11** **Cellular uptake behavior of a-LDH@356-PEG + US**

4T1 cells (1×10^6^ cells/well) were seeded in a petri dish (10 cm) and cultured overnight, followed by incubation with control (PBS), YW3-56 (2.8 μg mL^−1^), a-LDH-PEG (4 μg mL^−1^), a-LDH@356-PEG (4 μg mL^−1^), control + US, a-LDH-PEG + US (4 μg mL^−1^), and a-LDH@356-PEG + US (4 μg mL^−1^) for 24 h, respectively. US irradiation (40 kHz, 3W·cm^−2^) was performed at the 4^th^ h for 3 min. After rinsing with PBS, the cells were collected and digested overnight with nitration solution. Cobalt content was detected using an inductively coupled plasma mass spectrometry (ICP-MS, 710-ES, Varian, USA). For TEM analysis of a-LDH@356-PEG + US, the cells were fixed with 25% glutaraldehyde solution for 30 min at 25 °C away from light. Subsequently, embedded sections were performed for observation by a transmission electron microscope (JEM-2100, Japanese Electronics Company, Japan).

**3.12** **4T1 xenograft tumor model**

The experimental animal BALB/c female mice were bought from Beijing Vital River Laboratory Animal Technology Co., Ltd and received humane care. All animal experiments were conducted in accordance with the protocols approved by the Institutional Animal Care and Use Committee of Capital Medical University with the ethics number of AEEI-2018-174.

Female BALB/c mice (6−8 weeks old) were subcutaneously injected with luciferase-labeled 4T1 cells suspension (0.1 mL, 1×10^7^ cells/mL) into the third pair of breast pads on the left side.^[1]^ When the size of the tumor reached 75−100 mm^3^, mice were randomly divided into 7 groups (8 mice per group), which were intravenously injected with control (normal saline), YW3-56 (0.35 mg mL^−1^), a-LDH-PEG (0.5 mg mL^−1^), a-LDH@356-PEG (0.5 mg mL^−1^), control + US, a-LDH-PEG + US (0.5 mg mL^−1^), and a-LDH@356-PEG + US (0.5 mg mL^−1^) every two days, respectively. US irradiation (40 kHz, 3W·cm^−2^) was performed at 4 h post-injection for 3 min. During the treatment, the tumor volume (V=L×W^2^/2) and the body weight were measured every two days. After the mice were sacrificed on the 16^th^ day, the tumor tissue and major organs were harvested and weighed, and then frozen in liquid nitrogen or fixed in formalin for further use. Serum ALT, AST, UREA, CREA-S were measured with the corresponding kits by an automatic biochemical analyzer (BS-600, mindray). Complete blood counts were analyzed by an automatic blood cell analyzer (URIT-5160Vet, mindray).

**3.13** **Live imaging of small animals**

The tumor-bearing mice were intraperitoneally injected with 0.1 mL of *D*-Luciferin potassium salt (10 mg mL^−1^) at the 1^st^ day and 15^th^ day of administration. The mice were anesthetized with isoflurane 10 min after injection, then the fluorescence signals were obtained by the IVIS spectral system, and the data were processed and analyzed using Living Image 4.4.

**3.14** **Histopathological analysis and** **immunohistochemistry**

Immediately after removal, the major organs and tumors were fixed with 4% paraformaldehyde, and subsequently dehydrated and embedded in paraffin. After dewaxing and rehydrating, 2-µm-thcik sections were prepared and treated with hematoxylin and eosin (H&E) for routine examination. In terms of immunohistochemistry analysis, 4-μm-thcik sections were prepared, and then blocked and permeabilized in 2% BSA and 0.2% Triton X-100 in PBS at 25 °C for 20 min. The staining was conducted using the indicated combination of primary antibodies at 4 °C overnight, followed by treatment with secondary antibody (Alexa Fluor®568) for 1 h and DAPI for 5 min away from light. The sections were imaged by a section scanner (Pannoramic MIDI, 3DHISTECH, Hungary) and analysis by Image J.

**3.15 Single-cell flow mass spectrometry**

Fresh tumor tissues were prepared into single-cell suspensions using type IV collagenase and DNase. Live cells were isolated and fixed with FIX I (Fluidigm, CA, USA) solution for 15 min. The cells were stained with antibodies to surface markers with metal markers for 30 min, then infiltrated with Perm-S (Fluidigm, CA, USA), and stained with antibodies to intracellular markers and washed three times. After treating with iridium-containing DNA intermediates (191 Ir/193 Ir, final concentration 125 nM) in FIX and Perm (Fluidigm, CA, USA) solutions at room temperature for 1 h, they were re-suspended in 10% EQ 4 element calibration beads (Fluidigm, CA, USA) solution and then detected on Helios flow mass spectrometry (Fluidigm, CA, USA).


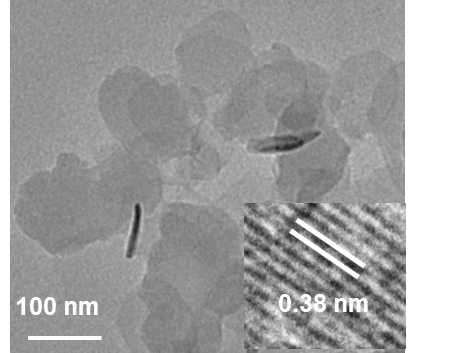


**Figure S1.** TEM image of CoW-LDH nanosheets.


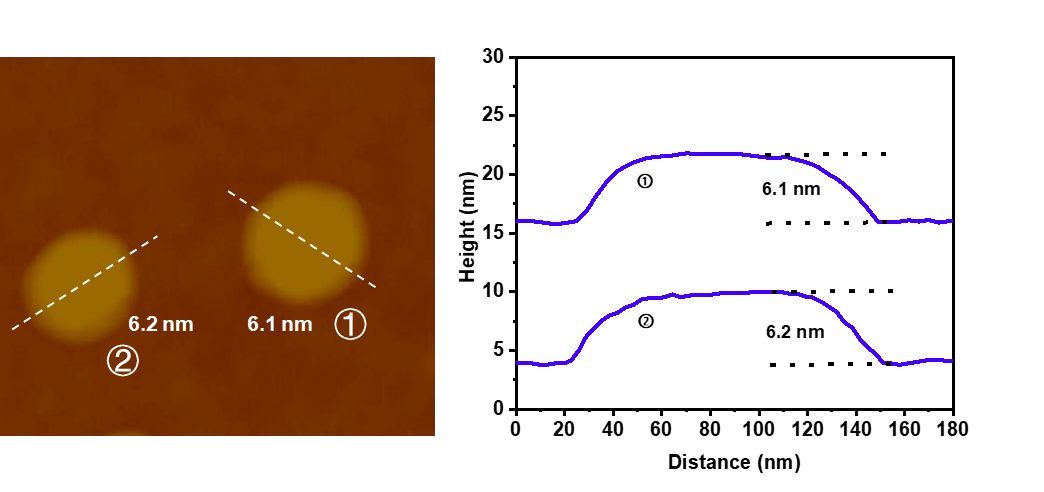


**Figure S2.** The AFM height image of a-LDH@356 nanosheets and its corresponding height profiles measured from 2 nanosheets.

**Figure S3.** UV-vis spectra of YW3-56, a-CoW-LDH and a-LDH@356 nanosheets.

**Figure S4.** Standard curve of the absorbance of YW3-56 (at 253 nm) *vs* concentration.

**Figure S5.** UV-vis spectra of DPBF in the presence of pure YW3-56 and a-CoW-LDH nanosheets under US irradiation (40 kHz, 3 W cm^−2^).

**Figure S6.** UV-vis spectra of ABDA in the presence of pure YW3-56 and a-CoW-LDH nanosheets under US irradiation (40 kHz, 3 W cm^−2^). (c) Normalized absorbance of ABDA in the presence of pure YW3-56, a-CoW-LDH and a-LDH@356 under US irradiation.

**Figure S7.** The fluorescence spectra of DCF in the presence of pure YW3-56, a-CoW-LDH and a-LDH@356 under US irradiation (40 kHz, 3 W cm^−2^).

**Figure S8.** DHR 123 probe sensing ·O_2_^−^ generation in aqueous for pure YW3-56, a-CoW-LDH and a-LDH@356 under US irradiation (40 kHz, 3 W cm^−2^).

**Figure S9.** (a) XPS survey spectra, (b) Co 2*p*, (c) O 1*s*, (d) W 4*f*, (e) C 1*s*, and (f) N 1*s* XPS spectra of a-LDH@356-PEG nanosheets.

**Figure S10.** Stability tests of a-LDH@356-PEG nanosheets in water, PBS and DMEM, respectively.

**Figure S11.** LC and EE of YW3-56 on a-CoW-LDH nanosheets after PEG modification (YW3-56: a-CoW-LDH = 2:1).


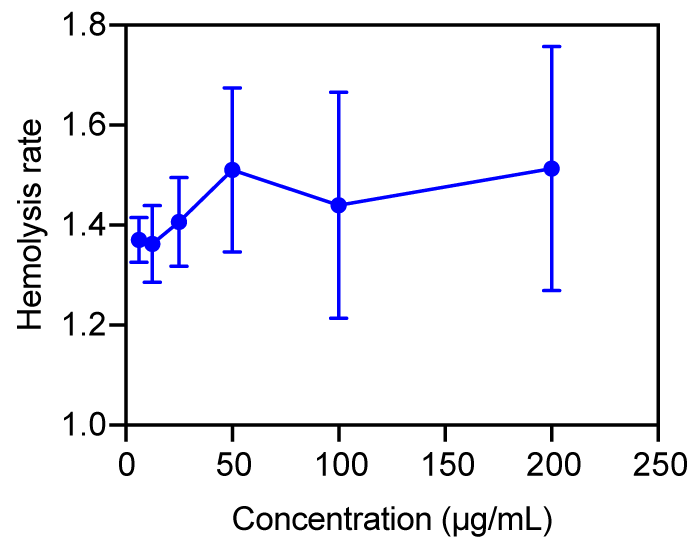


**Figure S12.** Hemolysis rate of a-LDH@356-PEG nanosheets.


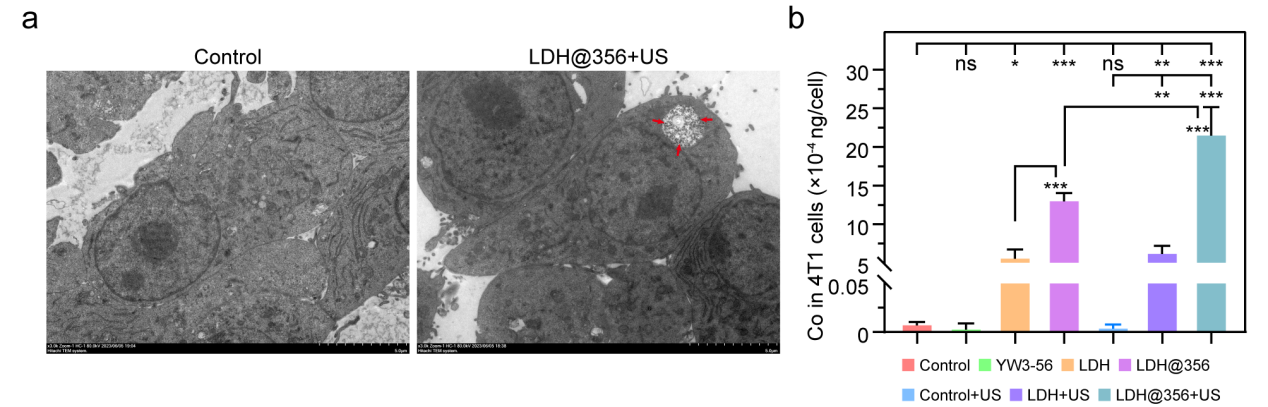


**Figure S13.** Cellular Uptake Behavior. (a) The uptake behavior of a-LDH@356-PEG + US in 4T1 cells observed by TEM. (b) The intracellular Co content determined by ICP-MS after different treatments. Data are presented as mean ± SD (n = 6). Statistical analysis was performed *via* one-way ANOVA. **p* <0.05, ***p* < 0.01, ****p* < 0.001.


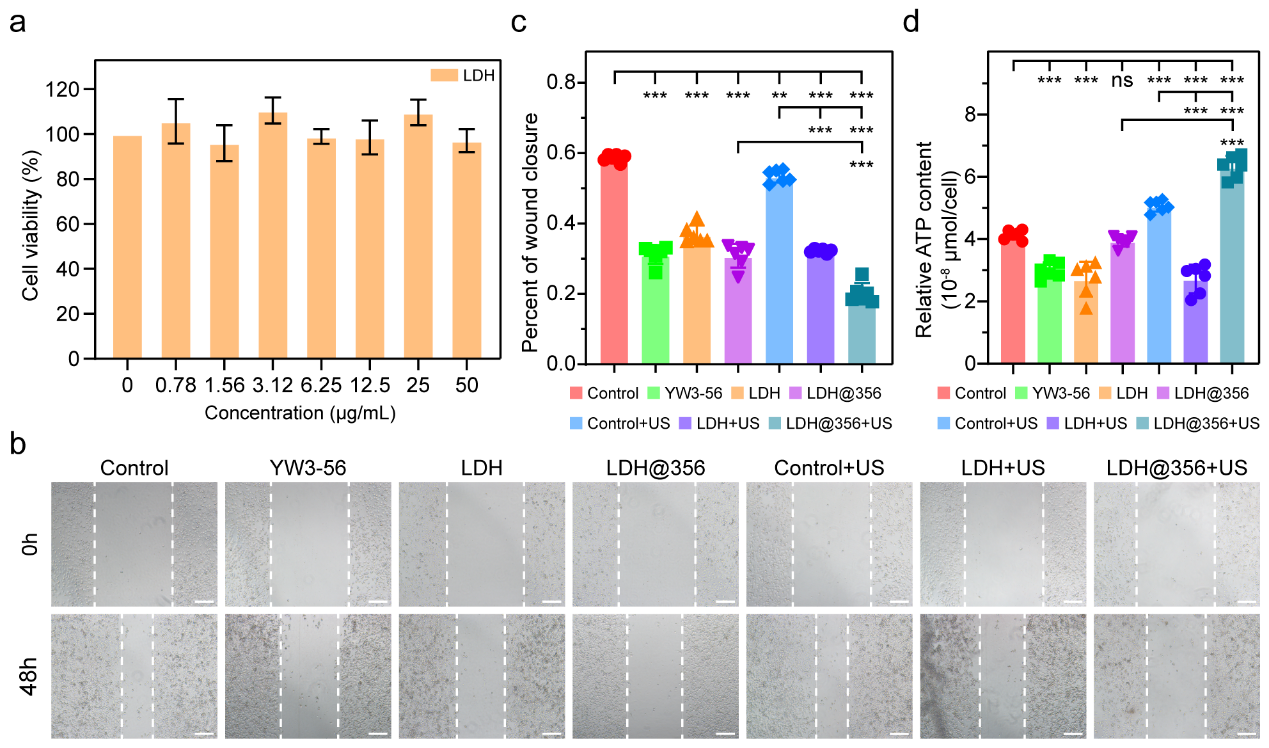


**Figure S14.** (a) Cell viability of 4T1 cells with a-LDH-PEG treatment at different concentrations. (b) Representative results of wound-healing assay and (c) corresponding quantitative analysis (Scale bar = 1000 μm). (d) Release of ATP from 4T1 cells after different treatments. Data are presented as mean ± SD (n = 6). Statistical analysis was performed *via* one-way ANOVA. ***p* <0.01, ****p* < 0.001.

**Table S1.** Cytotoxicity of YW3-56, a-LDH-PEG and a-LDH@356-PEG with or without US irradiation determined by MTT assay (IC_50_, μg mL^−1^).

| Compounds | Cytotoxic activity^a^ | Compounds | Cytotoxic activity^a^ |
| --- | --- | --- | --- |
| YW3-56 | 6.41 ± 0.21 |  |  |
| a-LDH-PEG | >10 | a-LDH-PEG +US | >10 |
| a-LDH@356-PEG | 7.66 ± 1.26 | a-LDH@356-PEG +US | 1.07 ± 0.49 |

^a^ Samples were tested in triplicate and presented as the mean ± SD.


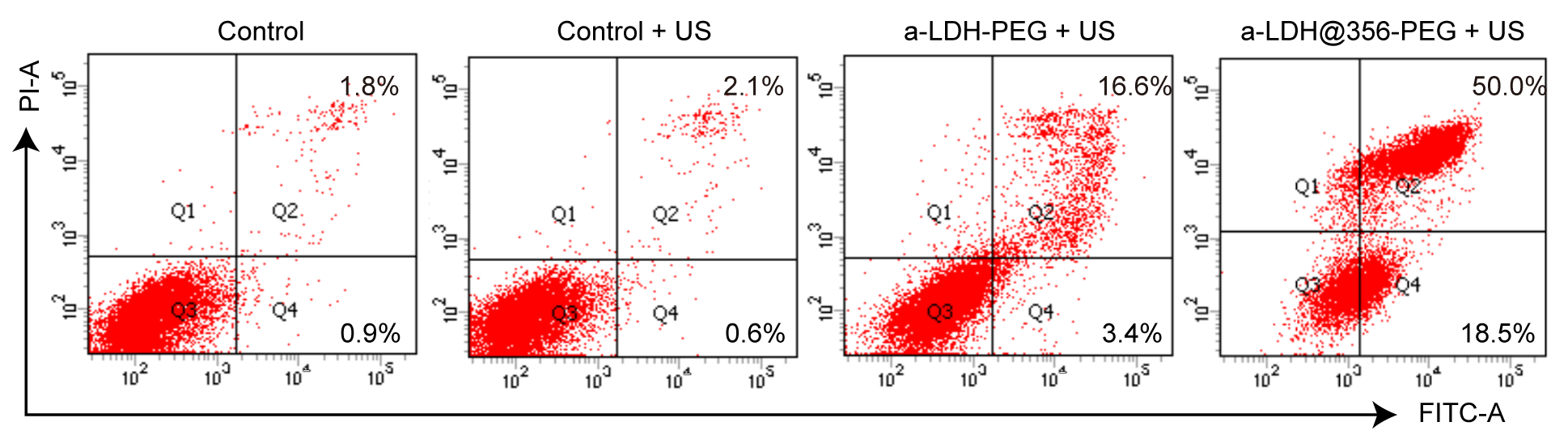


**Figure S15.** Cell apoptosis of control, control + US, a-LDH-PEG + US and a-LDH@356-PEG + US groups.


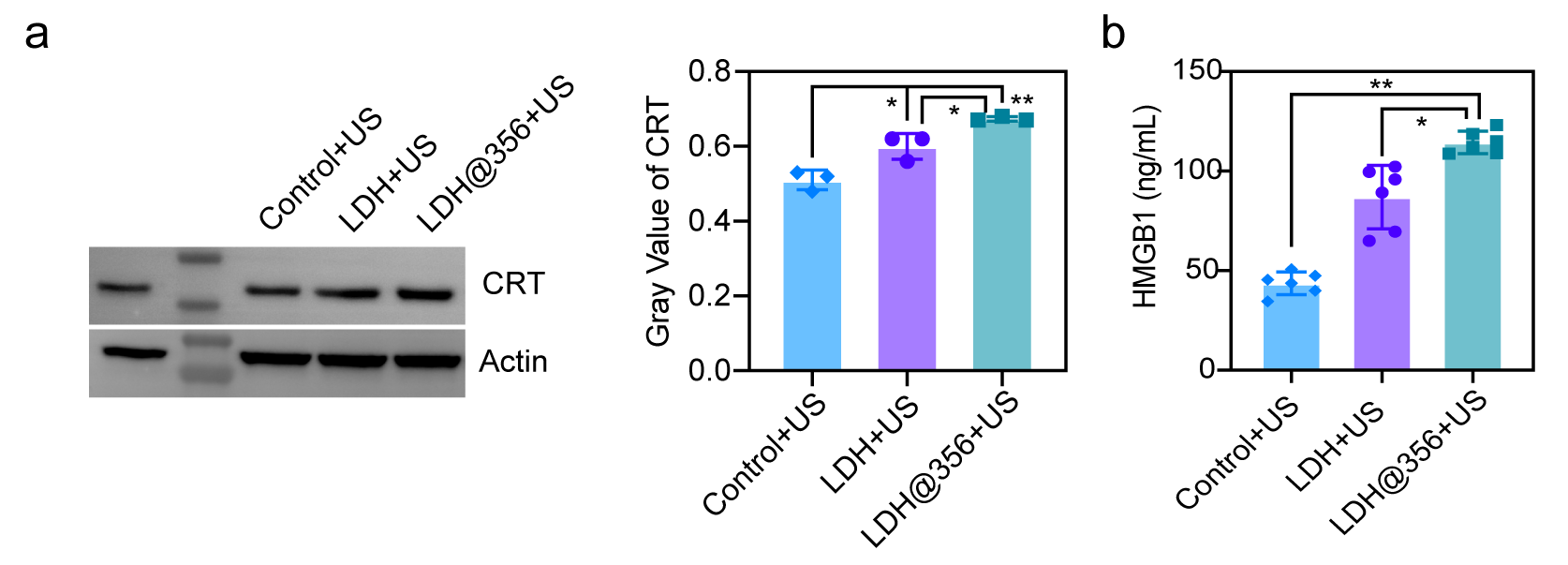


**Figure S16.** (a) CRT expression determined by western blot. (b) HMGB1 detected using ELISA.


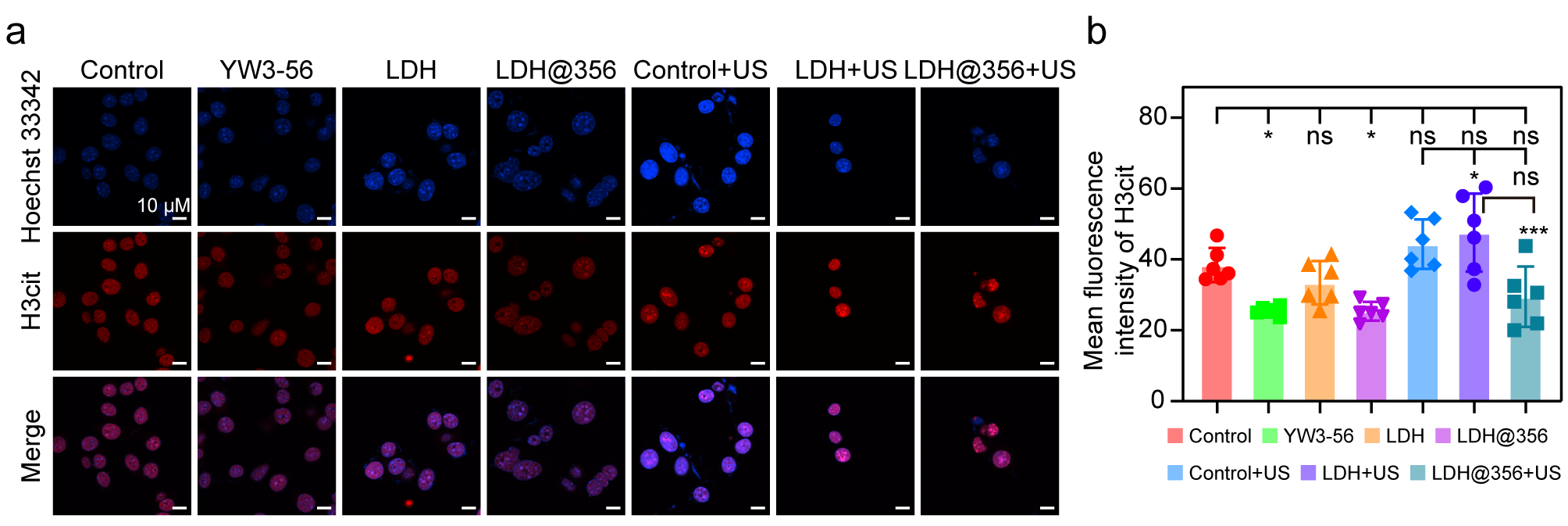


**Figure S17.** (a) H3cit staining images of 4T1 cells with different treatments and (b) corresponding quantitative analysis (Scale bar = 10 μm). Data are presented as mean ± SD (n = 6). Statistical analysis was performed *via* one-way ANOVA. **p* <0.05, ****p* < 0.001.

**
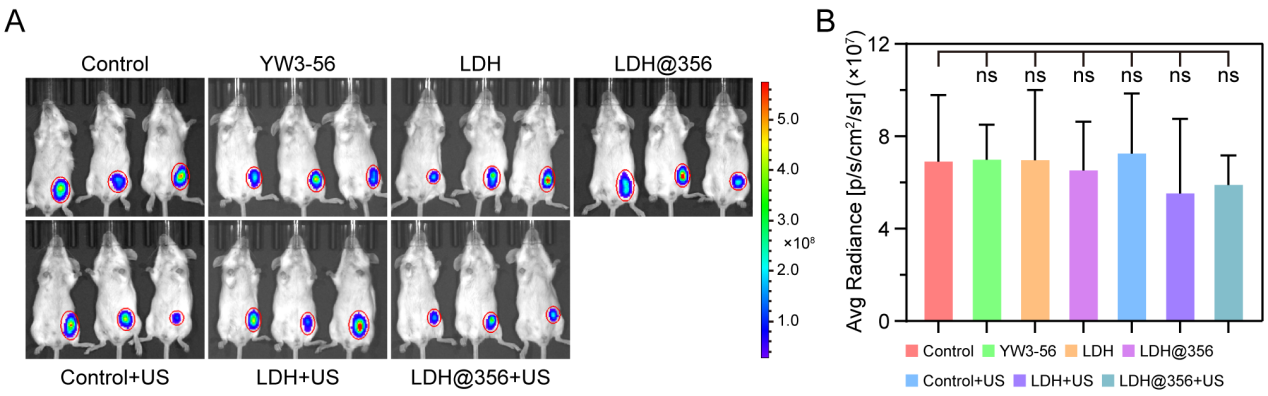
Figure S18.** (a) Living images of orthotopic 4T1 tumor-bearing mice on 1^st^ day of treatments and (b) corresponding quantitative analysis.


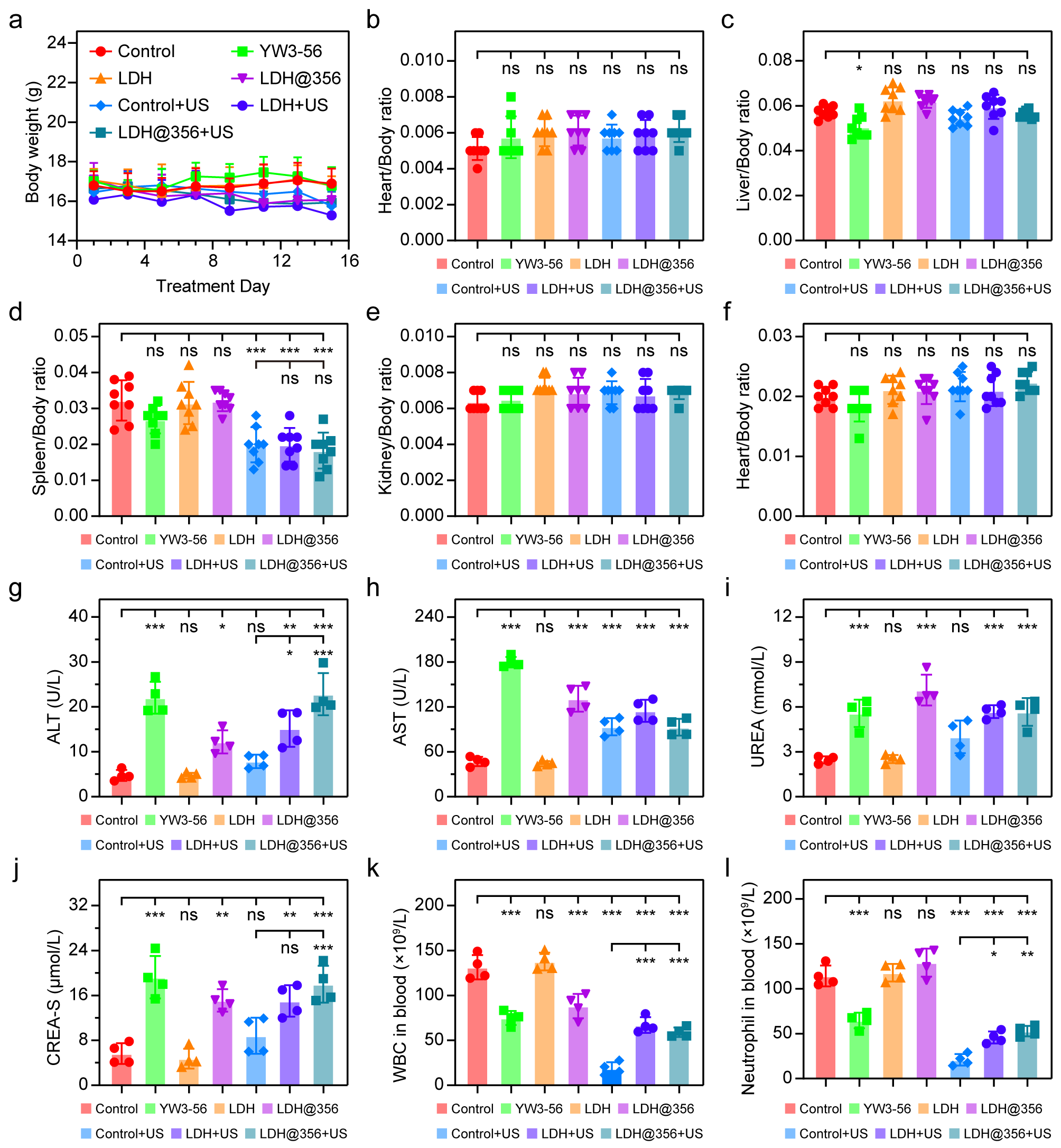


**Figure S19.** Biocompatibility evaluation of a-LDH@356-PEG. (a) Body weights of orthotopic 4T1 tumor-bearing mice during treatment. (b−f) Viscerosomatic ratio of major organs after different treatments. (g−j) The changes of serum biochemical indicators (ALT, AST, UREA, CREA-S) after different treatments. (k, l) The changes of blood cells after different treatments.


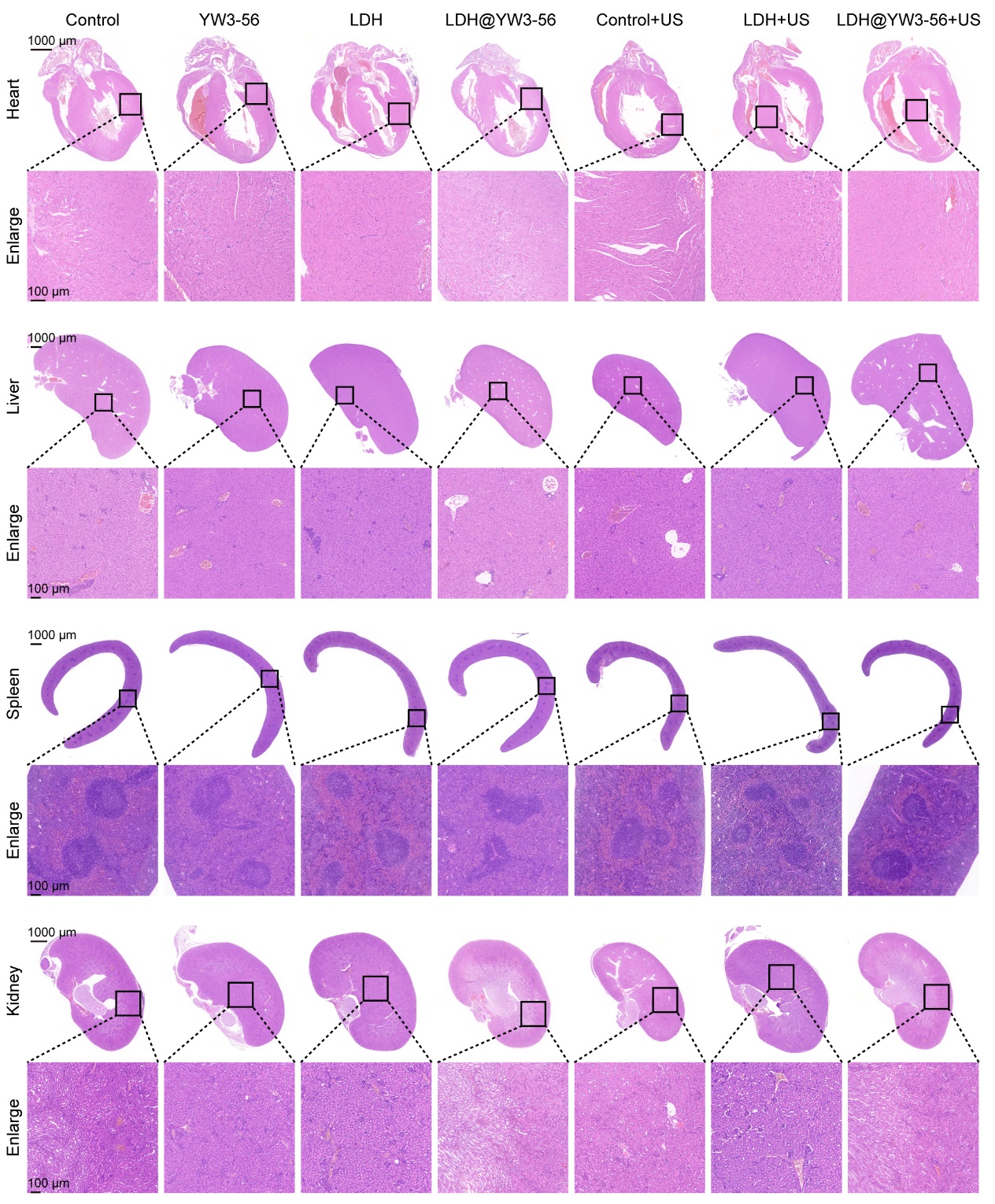


**Figure S20.** The H&E staining of major organ sections (heart, liver, spleen and kidney) after different treatments.


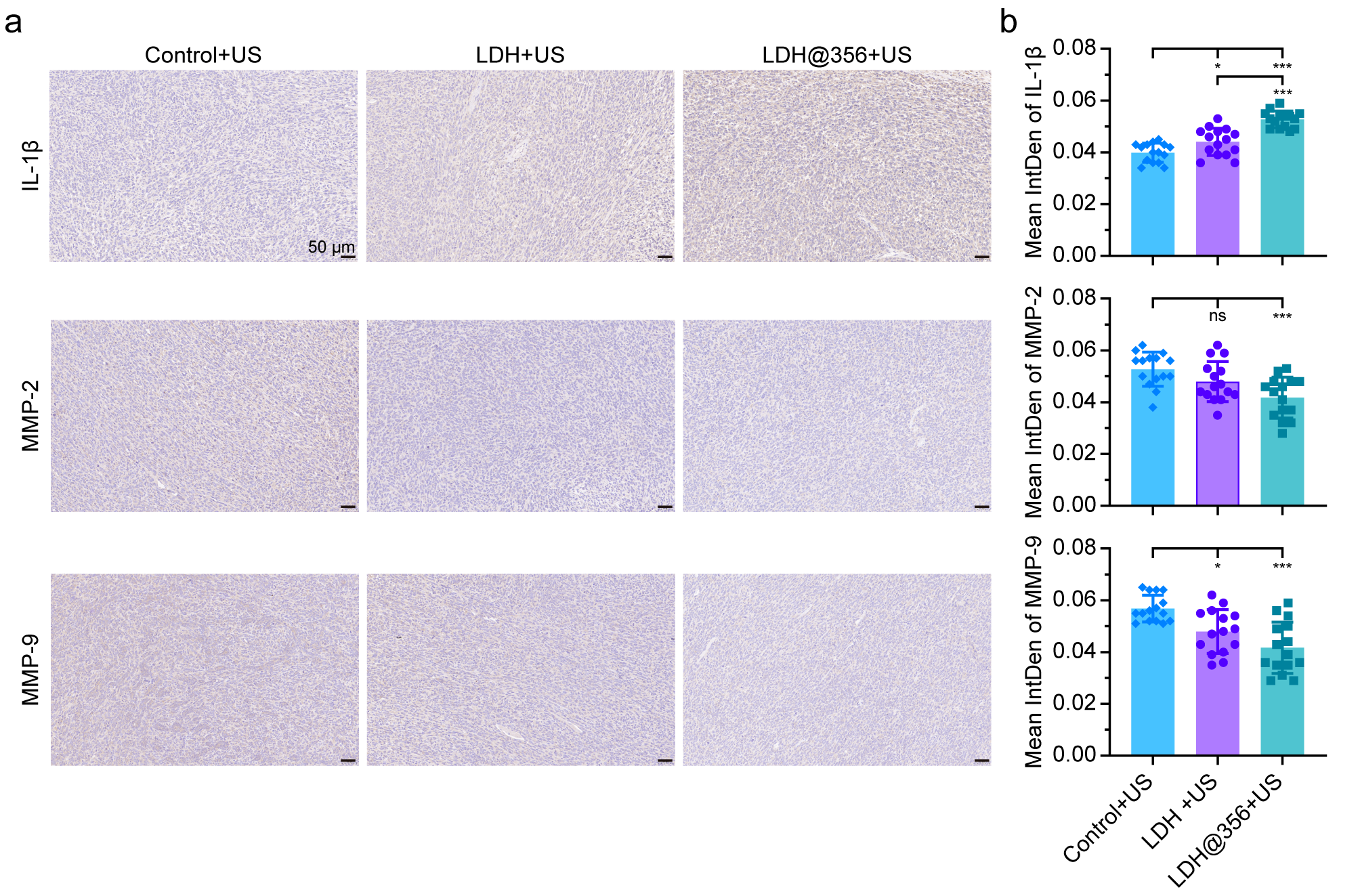


**Figure S21.** The expression of IL-1β, MMP2 and MMP9 determined by immunohistochemistry.


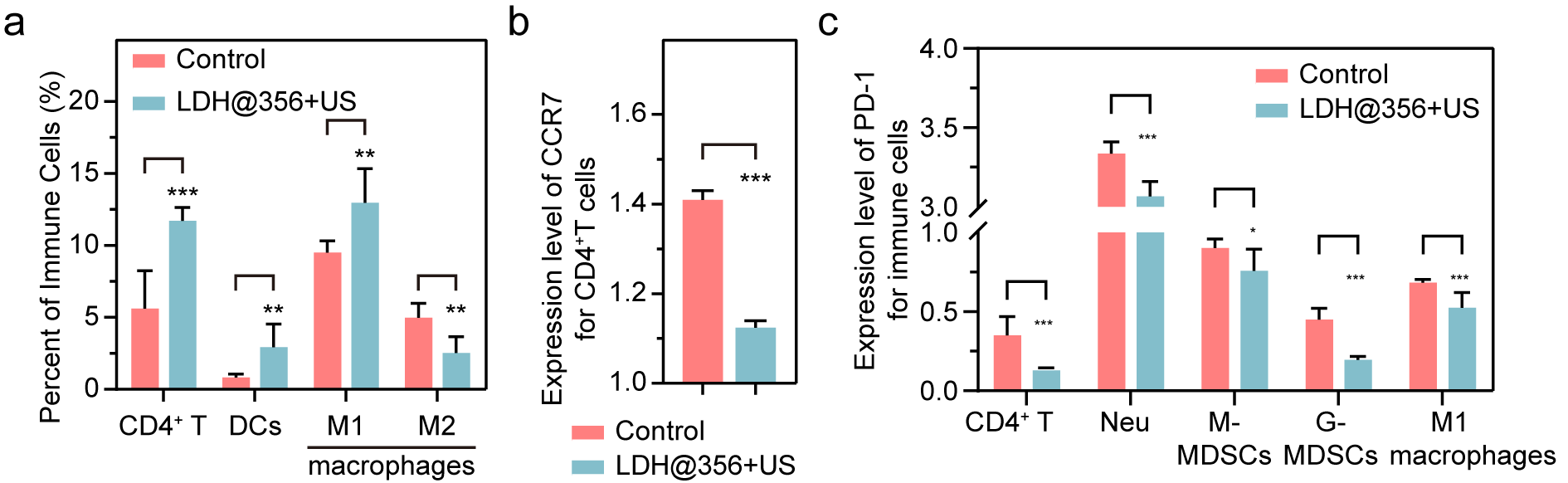


**Figure S22.** Effect of a-LDH@356-PEG + US on tumor immune microenvironment. (a) Proportion of CD4^+^ T cells, DCs, M1 and M2 macrophage cells in tumor tissues. (b) Expression level of CCR7 for CD4^+^ T cells. (c) Expression level of PD-1 for immune cells (e.g., CD4^+^ T cells, Neutrophil, M-MDSCs, G-MDSCs, M1 macrophages cells).

References

[1] Á. Teijeira, S. Garasa, M. Gato, C. Alfaro, I. Migueliz, A. Cirella, C. Andrea, M. C. Ochoa, I. Otano, I. Etxeberria, M. P. Andueza, C. P. Nieto, L. Resano, A. Azpilikueta, M. Allegretti, M. Pizzol, M. Ponz-Sarvisé, A. Rouzaut, M. F. Sanmamed, K. Schalper, M. Carleton, M. Mellado, M. E. Rodriguez-Ruiz, P. Berraondo, J. L. Perez-Gracia, I. Melero, *Immunity* **2020**, *52*, 856.
